# Pre-Machining of Rolled Plates as an Element of Minimising the Post-Machining Deformations

**DOI:** 10.3390/ma13214777

**Published:** 2020-10-26

**Authors:** Magdalena Zawada-Michałowska, Józef Kuczmaszewski, Paweł Pieśko

**Affiliations:** Faculty of Mechanical Engineering, Lublin University of Technology, 20-618 Lublin, Poland; j.kuczmaszewski@pollub.pl (J.K.); p.piesko@pollub.pl (P.P.)

**Keywords:** deformation, milling, thin-walled elements, aluminium alloys, pre-machining

## Abstract

The paper presents the influence of the milling strategy, the relation between the cutting tool feed direction and the rolling direction, as well as the pre-machining consisting of the removal of the textured surface layer of rolled plates in the rolling process on the thin-walled elements deformations made of the EN AW-2024 T351 wrought aluminium alloy, after milling. The research used strategies such as: high-performance cutting (HPC), high-speed cutting (HSC) and conventional milling (CM), as well as their combinations. Another tested variable was the relation between the tool feed direction and the rolling direction. In addition, the tests were carried out in the following versions: leaving the textured surface layer created after plastic working and with its removal with technological parameters corresponding to HSC and CM. Based on the obtained results, it was found that the post-machining deformation of thin-walled elements can be minimised owing to the use of a selected milling strategy and its combination with pre-machining (or lack thereof). It was also observed that larger deformations were obtained for samples after milling in the direction perpendicular to the rolling direction.

## 1. Introduction

A major problem in cutting of the thin-walled elements is post-machining deformation that occur when the workpiece is removed from the clamping device. The main reasons for their formation include, first of all, residual stresses generated at every stage of the technological process [1,2,3].

Residual stresses are defined as ‘the stresses remaining in the element after the external forces that cause its deformation ceased to act’. The literature also comprises terms such as locked-up stresses and internal stresses [4,5,6,7]. As a result of external factors, including, among others, mechanical, thermal, structural, or a combination thereof, the material deforms elastically and plastically. After removing the loads, the reversible changes disappear (elastic deformations), and the remaining irreversible changes (plastic deformations) cause the formation of residual stresses, counterbalancing each other within a specific area. This is mainly the result of an increase in the internal energy of the element leading to distortion of the crystal lattice. This brings about changes in material properties (e.g., reduction in corrosion resistance) and problems related to maintaining dimensional and shape accuracy. Residual stresses can also arise as a result of chemical and physicochemical interactions. In fact, the residual stresses are caused by mutually correlated factors (e.g., thermal and structural stresses occur after the quenching), and their exact distinction is exceedingly difficult [4,7,8,9,10].

Residual stresses appear in an element during surface cutting when the yield point of the material is exceeded. As a result, most manufactured machine parts have residual stresses that are remainder of cutting and/or assembly. Their nature and value change with the course of the technological process. They occur both in the surface layer and in the core. However, they usually reach the greatest values in the surface layer, especially in the textured zone, which is the part of the surface layer characterised by the preferential orientation of crystals or grains. The main parameters characterising residual stresses include: sign (tensile stresses—plus sign, compressive stresses—minus sign), value, gradient and depth of distribution [7,11,12,13,14].

Residual stresses can have both a positive and a negative impact on the operation of the manufactured element. In general, compressive residual stresses are beneficial because they increase creep resistance, fatigue corrosion resistance and strength as well as prevent microcracks. The opposite is true for tensile residual stresses, which lower the fatigue resistance and can cause intercrystalline corrosion. Depending on the analysed case, the aim is to create residual stresses with a specific sign or to remove them [9,15,16,17,18,19,20].

The most common models of residual stresses formation in the surface layer during cutting are the following: mechanical and thermal [21,22].

The mechanical model assumes the development of residual stresses in the surface layer as a result of cutting force (the influence of other factors, e.g., temperature, is negligible). It causes tensile stresses σ > 0. At a certain depth tensile stresses exceed the yield point of the material σ > R_e_ and elastic and plastic deformations appear (at greater depths, only elastic deformations occur). When the cutting blade is already in a different place, the cutting force in the analysed cross-section stops working, and the elastically stretched zone relaxes and leads to compression of the plastically deformed layer. The return to the original state is not possible. The final stress distribution is characterised by compressive stresses σ_1_ in the zone closer to the material surface, and tensile stresses σ_2_ in the deeper zone. The mechanical model corresponds mainly to cutting [21,22,23].

The thermal model assumes a significant influence of temperature and a negligible effect of cutting force. In the area of contact between the cutting tool and the workpiece, heat is generated and a temperature field is created. The result is an expansion of the material, limited by the cold core. Compressive stresses appear in the layer closer to the workpiece surface, while in the deeper layer, counterbalancing tensile stresses appear. When the yield point R_e_ is exceeded by compressive stresses, layer to a certain depth undergoes plastic deformation. If the heat source passes the tested cross-section, the material cools down, its outer layer shrinks and stresses are reduced. The return to the original state is impossible. In a cold state, tensile stresses σ_1_ occur in the proximal zone and compressive stresses σ_2_ occur in the deeper zone. The thermal model is characteristic of abrasive machining and HSC [21,22,23].

There is also a structural-volume model that results from the different specific volume of individual structural components of the material, and it plays an important role in the cutting of austenitic steels [21,22].

The residual stresses arising during the cutting process depend on many factors, including: [7,10,12,24,25,26]:

- Technological parameters, i.e., depth of cut, feed, cutting speed;
- Cutting tool geometry;
- Cooling conditions;
- Properties of the machined material;
- Degree of the cutting tool wear.

It is important that residual stresses can add up or subtract with the stresses caused by the action of external forces, both at the stage of further manufacturing of a given element and during its operation. The value of the resulting stresses may exceed the yield point of the material and cause, among others, non-uniform plastic deformation, loss of stability, distortion, twisting and other defects [7,18].

The industry is looking for technological guidelines that would help eliminate current methods of residual stress relaxation. The authors of the paper [27] indicated that the relaxation of residual stresses can be accelerated using thermal [28], mechanical, electrical [29] and magnetic [30] methods. A widely used method of reducing residual stresses is relief annealing. The disadvantage of this solution is mainly the high-cost intensity, especially in the case of large-size parts. Additionally, it changes the mechanical properties of the heat-treated element. One of the most effective methods of stabilising residual stresses is natural seasoning. Because of the fact that it is a long-term process, it is also of limited use and is more and more often replaced by vibration methods involving the removal of residual stresses by resonant vibration. However, the use of vibration methods for parts with significant size also generates additional costs. Stress relaxation of large-size components is quite troublesome, mainly because of decrease in production efficiency and energy losses. Often, during the relaxation process, the objects are destroyed, which is also associated with additional costs [7,15,18,31,32].

However, it should be noted that deformations of thin-walled elements also arise from other factors, such as temperature, clamping force, cutting force, tool geometry, etc., [33,34,35,36,37,38].

Based on the analysis of models of residual stress formation during cutting, including in particular the possibility of parallel occurrence of a mechanical model in combination with a thermal model, it can be assumed that a right selection of a milling strategy may bring beneficial results in terms of minimising deformations of thin-walled elements after milling. In addition, it is likely that the application of a pre-machining to remove the textured surface layer after rolling will reduce post-machining deformations.

The aim of the study is to determine the impact of the pre-machining application, consisting in the removal of the textured surface layer, on the minimisation of deformations of thin-walled elements, made of EN AW-2024 T351 aluminium alloy, after milling.

## 2. Materials and Methods

**Figure 1:**
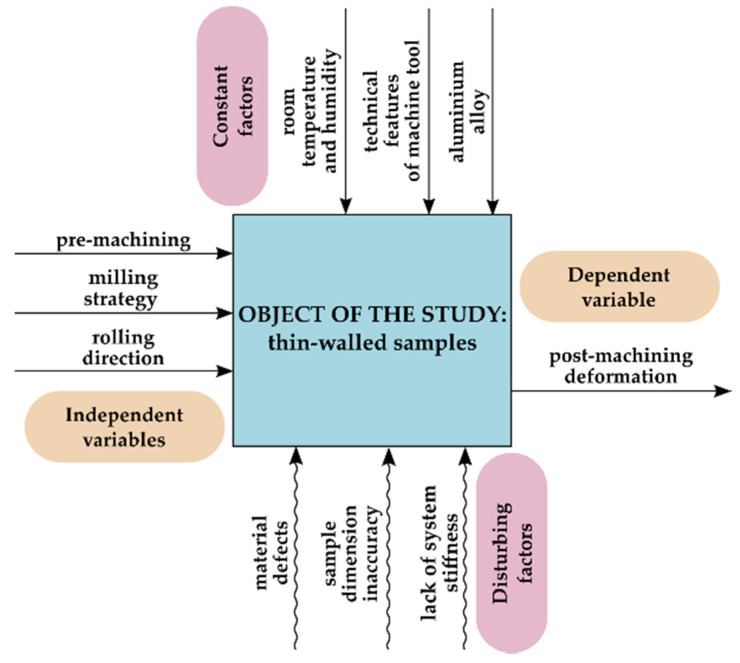
Heuristic model of research object.

The heuristic model of the research object with tested independent and dependent variables, as well as constant and disturbing factors is presented in Figure 1.

Thin-walled samples were defined as the research object. The independent variables were milling strategy, rolling direction and pre-machining, while the dependent—post-machining deformation. The constant factors include the technical features of the machine tool, temperature and humidity of the laboratory room as well as the type of material (in the analysed case, aluminium alloy). The disturbing factors were material defects, dimensional inaccuracy of samples and lack of system stiffness (machine tool, clamping device, workpiece, tool).

The following milling strategies were used in the research:

- High-performance cutting;
- High-performance cutting and conventional milling (CM);
- High-performance cutting and high-speed cutting;
- High-speed cutting;
- High-speed cutting and conventional milling (CM).

A characteristic feature of high-speed cutting, in comparison to conventional cutting, is an increase in the cutting speed v_c_, depending on the type of machined material. It is assumed that high-speed cutting begins when the cutting force decreases noticeably with increasing cutting speed v_c_. The use of HSC allows to reduce the main cutting time (even by more than 30%), increase the removal efficiency, reduce the cutting force and obtain the better quality of the machined surface. High-performance cutting is characterised by an increased volume of removed material per unit time during the cutting process. HPC assumes the maximum use of the spindle power with an increase in removal efficiency and a reduction of auxiliary times, resulting from an increase in positioning speed and shortening the tool change time. Comparing HPC with HSC, it should be noted that during HPC, higher values of the depth of cut a_p_, the milling width a_e_ and the feed per tooth f_z_ are used, but the cutting speed v_c_ is lower. On the other hand, HSC is characterised by higher cutting speed v_c_ and smaller section of the cutting layer. The effect of the presented differences is the application of both techniques. Generally, high-speed cutting is used for finishing, while high-performance cutting for roughing [39,40].

As an effect of the so-called ‘technological heredity’, another analysed independent variable was adopted, i.e., the relation between the cutting tool feed direction and the rolling direction:

- Tool feed direction perpendicular to the rolling direction (perpendicular direction);
- Tool feed direction parallel to the rolling direction (parallel direction).

In addition, the experiment was carried out in two configurations, removing and leaving the textured surface layer formed after plastic working. Pre-machining was performed with technological parameters corresponding to HSC and CM.

The tests were conducted on samples made of the EN AW-2024 T351 wrought aluminium alloy that is widely used, among others, in the aerospace industry. It is characterised by high strength and good machinability, but low corrosion resistance and limited weldability. The chemical composition and properties of the EN AW-2024 T351 aluminium alloy are shown in Table 1.

**Figure 2:**
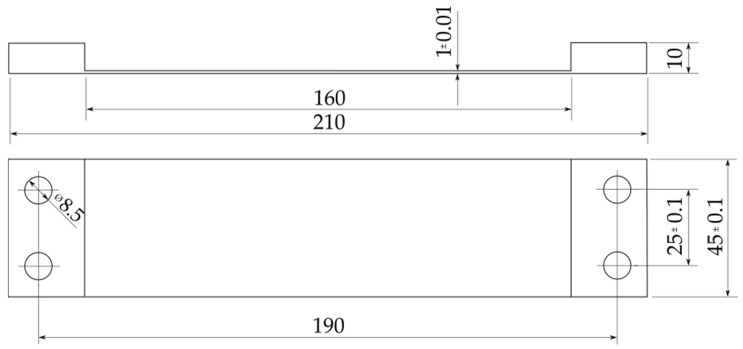
The construction drawing of the sample after milling.

Figure 2 presents the construction drawing of the sample after milling. The samples were made of 10-mm thick rolled plate. The final thickness of the thin-walled bottom was 1 mm, while the width and length of the milled pocket were 45 mm and 160 mm, respectively. The holes ensure uniform clamping conditions. The adoption of such a solution made it possible to obtain repeatability of clamping during tests and the influence of the clamping force on the tested deformations was eliminated.

**Figure 3:**
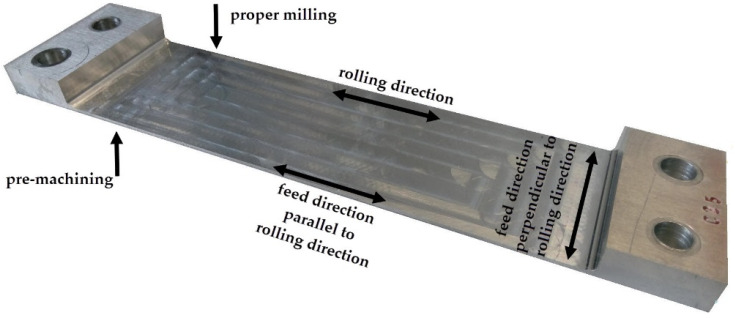
View of the sample with marking the relation between cutting tool feed direction and rolling direction.

A view of the sample with a pocket along the entire width and marking the relation between cutting tool feed direction and rolling direction as well as the machined surfaces during the pre-machining and the proper milling is presented in Figure 3. 

**Figure 4:**
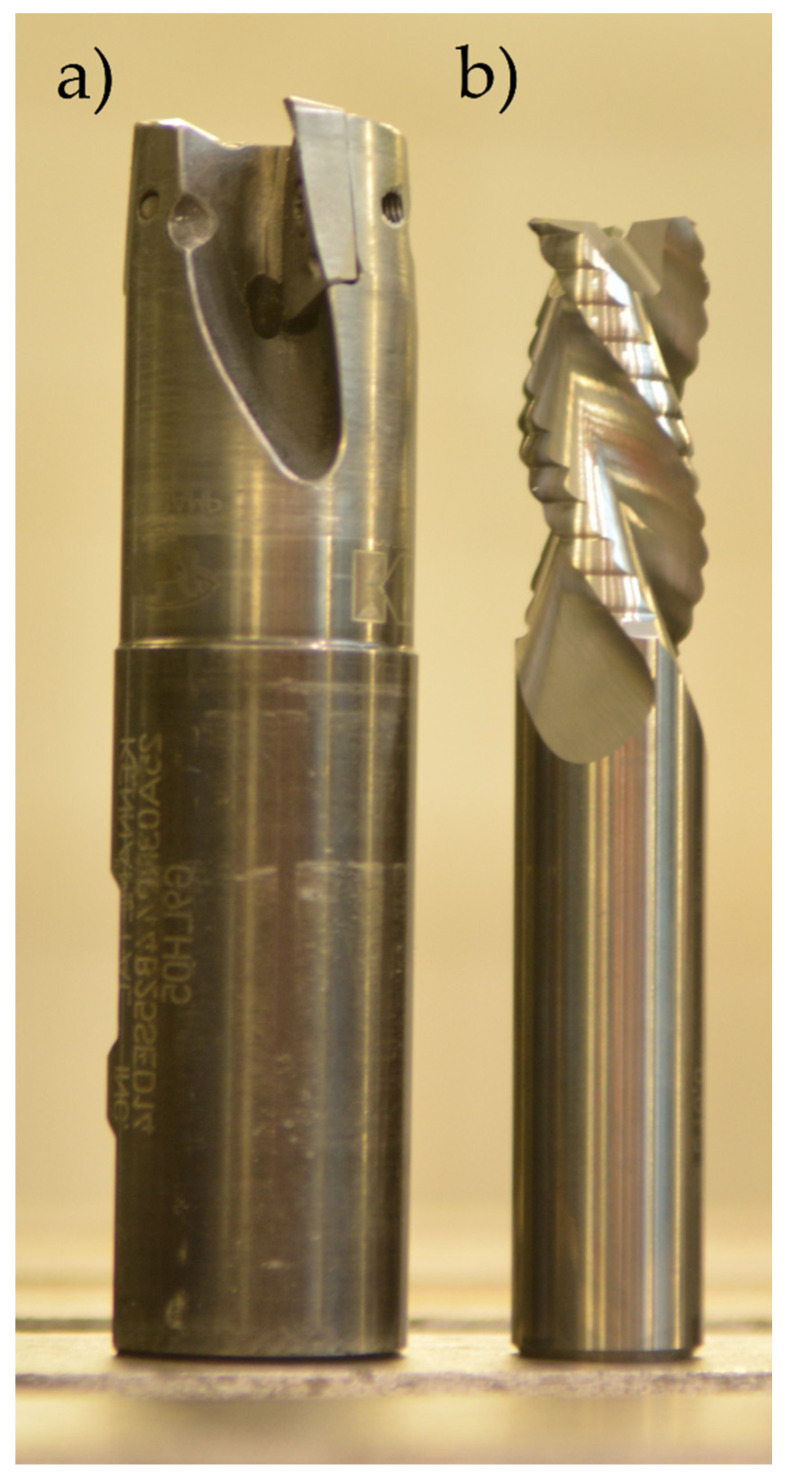
Tools used: (**a**) Kennametal indexable milling cutter, (**b**) Sandvik monolithic milling cutter.

The research used an Avia VMC 800 HS vertical machining centre (FABRYKA OBRABIAREK PRECYZYJNYCH AVIA S.A., Warsaw, Poland) and the following tools:

- Kennametal indexable milling cutter (25A03R044B25SED14) with adapted cutting inserts (EDCT140416PDFRLDJ) (Kennametal, Pittsburgh, PA, USA), material: KC410M—a carbide coated with TiB2 protective coating by PVD method—used for high-performance cutting (Figure 4a);
- Sandvik monolithic milling cutter (R216.33-16040-AC32U) (Sandvik, Stockholm, Sweden), material: H10F—a tungsten carbide without any protective coating—used for high-speed cutting and conventional milling (Figure 4b).

Technical parameters of the cutting tools used are summarised in Table 2.

The technological parameters corresponding to the individual milling strategies are presented in Table 3. It is worth noting that the selection of values was based on the manufacturers’ recommendations and the authors’ own research.

The tests were carried out in two versions, removing and leaving the textured surface layer formed after rolling. The thickness of the removed layer was the result of the research, which was presented in more detail in [45]. As part of a previously defined strategy, surfaces were machined according to Figure 3. Pre-machining was carried out with two ranges of technological parameters, corresponding to CM, a_p_ = 0.4 mm, v_c_ = 200 m/min, f_z_ = 0.02 mm/tooth and HSC, a_p_ = 0.4 mm, v_c_ = 1200 m/min, f_z_ = 0.02 mm/tooth.

The cutting programs were generated in the NX10 software by Siemens, in which the kinematics of the milling process was also simulated, and the possibility of collisions was eliminated.

**Figure 5:**
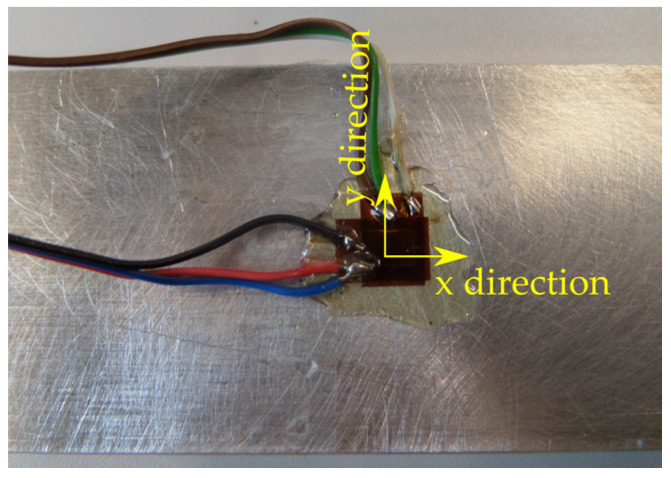
Foil strain gauges glued to the surface of the sample.

Deformation measurements were made applying the strain gauge method with the use of individual Tenmex TF-5-2x foil strain gauges (Tenmex, Łodź, Poland), which were glued according to Figure 5 (x direction–longitudinal strain gauge, y direction–transversal strain gauge). The standard surface preparation procedure of the samples, the LOCTITE 401 cyanoacrylate adhesive (Henkel, Düsseldorf, Germany) and the protective M-COAT A AIR-DRYING POLYURETHANE COATING layer (Micro-Measurements, Wendell, NC, USA) were used. Table 4 provides the technical specification of Tenmex TF-5-2x foil strain gauges.

**Figure 6:**
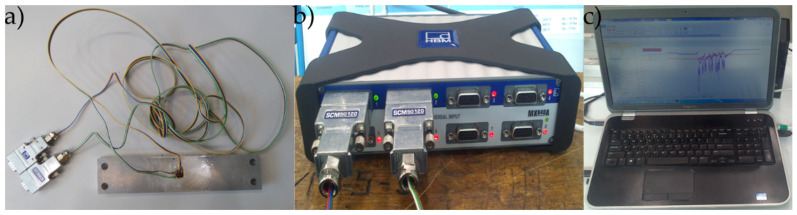
Measurement of deformations using the strain gauge method: (**a**) sample with connected SCMSG120 adapters, (**b**) HBM 1-MX840A amplifier, (**c**) computer with CatmanEasy V35.1 DAQ Project software.

The deformation measurement was carried out using strain gauges connected with soldered cables to the SCMSG120 adapters (Figure 6a), the HBM 1-MX840A universal measuring amplifier (HBM, Darmstadt, Germany) (Figure 6b), the optoelectronic link and the computer with CatmanEasy V35.1 DAQ PROJECT software (Figure 6c).

**Figure 7:**
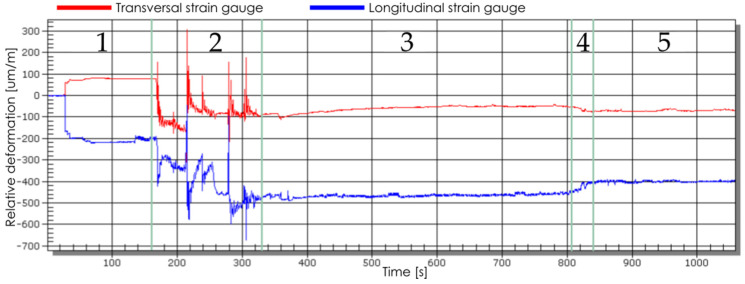
An exemplary course of the change of the relative deformation value in time: 1—fastening the sample in the clamping device, 2—the phase of the influence of disturbing factors, 3—milling process, 4—unfastening the sample from the clamping device, 5—stabilisation.

In the recorded courses of changes in the value of the relative deformation (unit: µm/m) over time (unit: s), five phases were distinguished (Figure 7):

1. Fastening the sample in the clamping device;
2. The phase of the influence of disturbing factors (e.g., turning on the coolant oil);
3. Milling process;
4. Unfastening the sample from the clamping device;
5. Stabilisation.

The value and sign of deformations obtained in the stabilisation phase (after the sample temperature had been determined) were taken into account. The tests were repeated 5× for each analysed configuration.

## 3. Results

**Figure 8:**
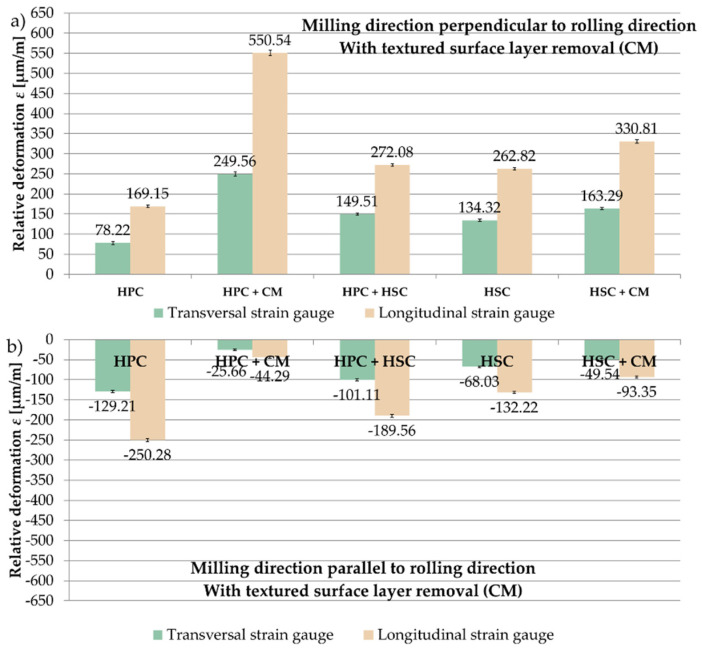
Relative deformation ε obtained for samples in which the textured surface layer (CM) was removed, the analysed cutting strategies and the relation of the milling direction to the rolling direction: (**a**) perpendicular, (**b**) parallel.

The analysis of the results of strain gauge tests began from samples in which the textured surface layer formed after rolling with the use of CM was removed. Relative deformations ε obtained after milling in the perpendicular and parallel directions to the rolling direction and for the tested cutting strategies are presented in Figure 8. On the basis of the received results, it was found that the relative deformations ε were greater on longitudinal strain gauges. Relative deformations ε were recorded for the perpendicular milling to the rolling direction ε with the plus sign, while in the case of parallel milling, with the minus sign. For samples that were milled in perpendicular direction to the rolling direction, the greatest value of the relative deformation ε was obtained after combining HPC with CM, ε = 550.54 µm/m, and the smallest after HPC, ε = 169.15 µm/m (70% less for the combination of HPC with CM). For samples milled in parallel direction, the maximum value of the relative deformation ε occurred after HPC, ε = −250.28 µm/m, and the minimum after combining HPC with CM, ε = −44.29 µm/m (80% less compared to the HPC strategy).

**Figure 9:**
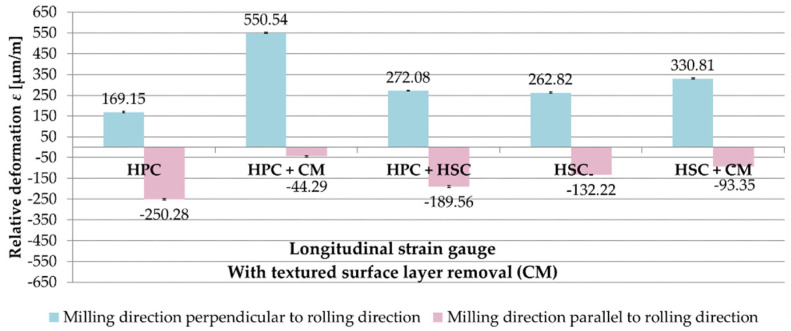
Comparison of relative deformations ε obtained on longitudinal strain gauges, depending on the relation of the milling direction to the rolling direction (samples with the removed surface layer—CM).

A comparison of relative deformations ε obtained on longitudinal strain gauges, depending on the relation of the milling direction to the rolling direction for samples with the removed surface layer (technological parameters for CM) is shown in Figure 9. Based on the received results, it was found that, the samples milled perpendicularly to the rolling direction underwent greater relative deformations ε than samples milled parallel to the rolling direction. It is also worth noting that the different signs of relative deformations ε were recorded for the analysed relations between the cutting tool feed direction and the rolling direction. A deviation was noted only with the HPC strategy, for which the relative deformation ε was almost 50% greater after parallel milling to the rolling direction compared to perpendicular milling. For the combination of HPC with CM, the relative deformation ε value was over 1100% greater for perpendicular milling to the rolling direction than for parallel milling. In the case of the combination of HPC and HSC, the difference was about 40%, while for the HSC and the combination of HSC with CM, it was 100% and 250% respectively (in relation to the parallel direction).

**Figure 10:**
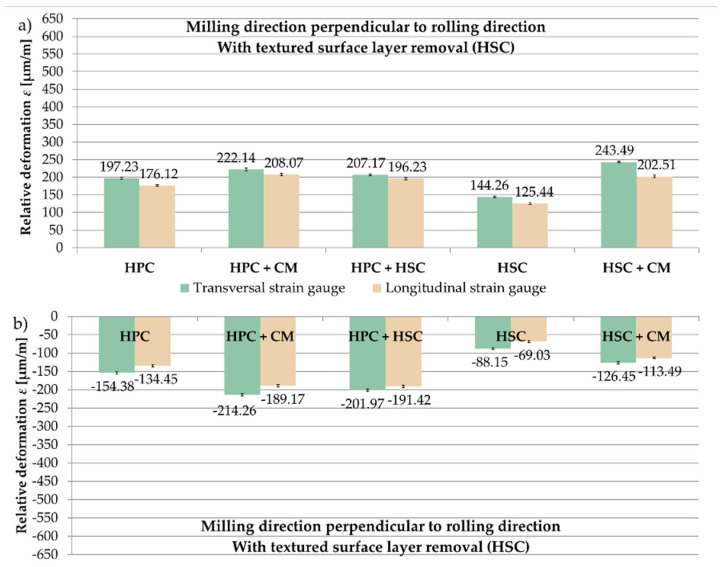
Relative deformation ε obtained for samples in which the textured surface layer (HSC) was removed, the analysed cutting strategies and the relation of the milling direction to the rolling direction: (**a**) perpendicular, (**b**) parallel.

The results for the samples with pre-machining with parameters corresponding to the HSC range were then analysed. Figure 10 presents the relative deformations ε obtained after milling in the perpendicular and parallel directions to the rolling direction and the examined cutting strategies. On the basis of the received results, it was observed that the recorded relative deformations ε were greater on transversal strain gauges. For samples machined perpendicularly to the rolling direction, the maximum relative deformation ε, on a longitudinal strain gauge, was obtained after a strategy combining HPC with CM, ε = 208.07 µm/m, and the minimum after HSC, ε = 125.44 µm/m (almost 40% less than for HPC with CM). For samples that were milled in parallel direction, the greatest value of the relative deformation ε, also on the longitudinal strain gauge, occurred after the combination of HPC and HSC, ε = −191.42 µm/m, while the lowest after HSC, ε = −69.03 µm/m (over 60% less compared to HPC with CM).

**Figure 11:**
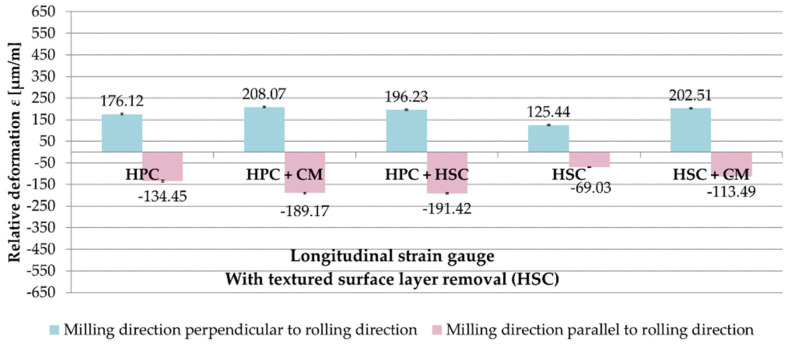
Comparison of relative deformations ε obtained on longitudinal strain gauges, depending on the relation of the milling direction to the rolling direction (samples with the removed surface layer—HSC).

Figure 11 shows a comparison of the relative deformations ε from longitudinal strain gauges, depending on the relation of the milling direction to the rolling direction for samples with HSC pre-machining. While analysing the obtained results, it was noted that the samples, after milling in the direction perpendicular to the rolling direction, underwent greater relative deformations ε than samples machined parallel to the rolling direction. In the case of HPC, HPC with CM as well as the combination of HPC and HSC, the differences were 30%, 10% and 3%, respectively (in proportion to the parallel direction). After HSC and strategy combining HSC with CM, the relative deformations ε were about 80% greater after cutting in the perpendicular direction in comparison to the parallel direction.

In order to compare the relative deformations ε in configurations with pre-machining with technological parameters corresponding to the HSC and CM as well as without its use, the results presented in [46] were referenced. Table 5 shows relative deformations ε obtained for samples without textured surface layer removal after milling in the perpendicular and parallel directions to the rolling direction and for the tested cutting strategies.

**Figure 12:**
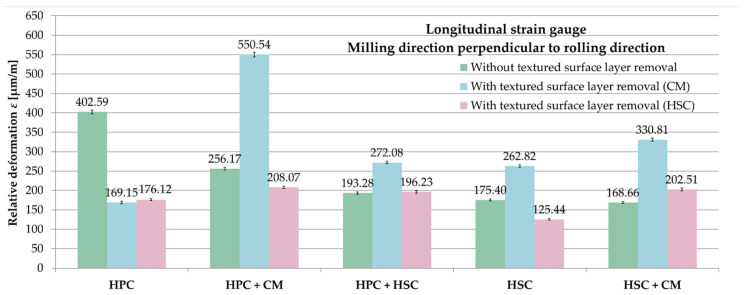
Comparison of relative deformations ε from longitudinal strain gauges depending on the applied pre-machining–milling direction perpendicular to rolling direction.

A list of relative deformations ε from longitudinal strain gauges, depending on the applied pre-machining, for samples milled perpendicularly to the rolling direction is shown in Figure 12. For comparative purposes, in the variant with leaving the textured surface layer after rolling, the absolute values of the obtained relative deformations ε were used. On the basis of the received results, it was noticed that removing the surface layer with parameters corresponding to CM does not reduce the relative deformation ε. A deviation was only noted with the HPC strategy. In this case, an approximately 4% difference between the relative deformations ε was obtained from two variants of pre-machining (CM and HSC parameters). In relation to these values, while leaving the textured surface layer, about 130% greater relative deformations ε occurred. For the combination of HPC with CM and HSC technique, greater relative deformations ε by about 20% and 40% were obtained by leaving a surface layer than by applying a pre-machining with HSC parameters. Combining HPC with CM, the relative deformations ε were reduced by 50% during leaving a textured surface layer than when removing it in the variant of CM. For HSC, there was a similar difference of 30%. Different dependencies occurred in the following configurations: HPC with HSC and HSC with CM. Relative deformations ε were lesser by 1.5% and over 15%, respectively, for the variant when leaving the surface layer than with its removal in the HSC range. In the case of the HPC with HSC and HSC with CM, the relative deformations ε were greater by 40% and almost 100% using CM pre-machining in comparison to the variant with leaving a textured surface layer.

**Figure 13:**
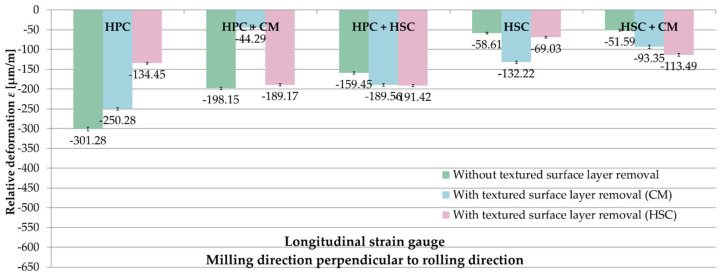
Comparison of the relative deformations ε from the longitudinal strain gauges depending on the applied pre-machining–milling direction parallel to rolling direction.

Figure 13 presents a comparison of the relative deformations ε from longitudinal strain gauges, depending on the applied pre-machining for samples milled parallel to the rolling direction. Analysing the obtained results, it was found that the influence of the selected pre-machining variant on the value of relative deformations ε, which are closely related to the milling strategy, cannot be unequivocally determined. All relative deformations ε were received with the tool feed parallel to the rolling direction with the minus sign. For HPC with HSC, HSC and combination of HSC with CM, the lowest relative deformations ε were noted when leaving a textured surface layer. In the case of HPC and HPC with CM, the greatest values were observed for the variant with no pre-machining. After HPC, the relative deformations ε were respectively 20% and over 120% greater during leaving the surface layer than when using pre-machining with the parameters of CM and HSC. With the strategy combining HPC with CM, the relative deformations ε were increased by 5% and almost 350% for samples where the surface layer was left in relation to its removal in the HSC and CM variants. For the combination of HPC with HSC, relative deformations ε were noted about 15% lower in the absence of pre-machining with regard to its use with the parameters corresponding to HSC and CM, respectively. Similar dependencies were obtained using HSC technique, with the differences being 15% and 55% compared to HSC and CM pre-machining. For the strategy combining HSC with CM, 45% and 55% lower relative deformations ε were obtained also for the variant with an unremoved surface layer than for HSC and CM pre-machining.

In conclusion, the possibility of reducing the relative deformations ε was found in thin-walled elements made of aluminium alloy EN AW-2024 T351, by using an appropriate cutting strategy in combination with the appropriate pre-machining variant. Additionally, it was observed that the samples milled perpendicularly to the rolling direction underwent greater relative deformations ε compared to samples milled in parallel direction.

The obtained results of the authors’ own research were subjected to statistical analysis aimed at assessing the significance of differences. The selection of the right statistical test is not easy and depends on many factors, including the type of trial (dependent, independent), the number of comparative groups (two or more) and the measurement scale (quantitative or qualitative). The procedure of comparing two trials with quantitative independent variables was realised. A significance level of α = 0.05, typical for scientific research, was assumed [47].

The statistical analysis was started with the examination whether the distributions of the analysed variables were subjected to the normal distribution. For this purpose, the Shapiro-Wilk test was used, on the basis of which it was found for all variables that their distributions were close to the normal distribution. In the next step, the hypothesis of equality of variances was verified with the use of Fisher-Snedecor distribution (F-distribution). Then the calculated value of the F statistic was compared with the critical value F_cr_ determined on the basis of the assumed significance level α and the numbers of degrees of freedom f_1_ and f_2_ determined for two samples. After confirming the null hypothesis of equality variances, the Student’s t-test was then applied to verify the hypothesis about the equality of mean values. The calculated value of the t statistic was compared with the coefficient t_cr_ read from the appropriate table determined on the basis of the adopted significance level α and the number of degrees of freedom f. In case of rejection of the null hypothesis of equality of variances, instead of the Student’s t-test, the Cochran-Cox adjustment should be used to test the hypotheses about equality of mean values (it was not used).

Table 6, Table 7 and Table 8 present the results of testing the hypothesis of the equality of variances, and the hypothesis of the equality of mean values for relative deformations ε in versions with leaving a textured surface layer and after its removal with the technological parameters corresponding to CM and HSC. Based on the performed verification, equality of variances was found in all cases. On the other hand, the test of the hypothesis on the equality of mean values showed significant differences, and the deviation was noted for only one variant (HSC pre-machining, comparison of HPC + CM and HPC + HSC, milling in the direction parallel to the rolling direction). The conducted statistical analysis allowed for the formulation of a conclusion about the significant influence of the cutting strategy on the relative deformations ε at the adopted significance level of α = 0.05. Because of the observation of clear differences between the relative deformations ε for the considered relations between the milling direction and the rolling direction, it was not necessary to check this configuration.

The statistical verification of the results of relative deformations ε for the tested variants of pre-machining is shown in Table 9.

It should be noted that statistical comparison of pre-machining variants was performed only for similar results, impossible to distinguish clearly. According to Table 9, equality of variances was observed in each case. Checking the hypothesis of the equality of mean values, significant differences were noted for the perpendicular direction and HPC technique (comparison of CM and HSC) and for the parallel direction and the strategy combining HPC with HSC (comparison of CM and HSC), as well as HSC (comparison of the lack of pre-machining and HSC). On the other hand, the equality of mean values was obtained for the perpendicular direction and the connection of HPC with HSC, and for the parallel direction and the combination of HPC and CM. The lack of pre-machining and its performance with the HSC parameters were compared. In other cases, which were not subjected to testing, it was clearly stated that they were different from each other. The statistical analysis allowed for the conclusion that the pre-machining variant affects the relative deformations ε at the adopted level of significance α = 0.05.

Summarising the results of relative deformations ε and statistical analysis, it was found that all the analysed variables, i.e., cutting strategy, the relation of the milling direction to the rolling direction, and the pre-machining (or lack thereof) influence the value of relative deformations ε.

## 4. Conclusions

The paper analysed the problem of deformations of thin-walled elements made of a selected aluminium alloy (EN AW-2024 T351) after milling. Based on the analysis of the literature and the authors’ own research, it was assumed that the appropriate strategy, together with pre-machining significantly affect the form and value of post-machining deformations of thin-walled elements made of aluminium alloys.

Based on the research and analysis of the obtained results, the following conclusions were drawn:

- The cutting strategy in combination with pre-machining (or lack thereof) consisting of the removal of the textured surface layer after rolling affects the relative deformation ε.
- The combination of HSC, HPC and CM has a positive effect on minimising the deformation of thin-walled elements after milling. Conventional milling, despite its relatively low efficiency, can have practical applications in the production of thin-walled elements.
- When manufacturing parts with a wall thickness of less than 2 mm, in particular less than 1 mm, removal of the rolled surface layer from the surface opposite to the machined surface, combined with the appropriate selection of a cutting strategy, can minimise post-machining deformations.
- It was found that milling direction in relation to the rolling direction on the relative deformation ε was influential. Therefore, the feed direction of the cutting tool should be parallel to the rolling direction.
- It is also necessary to remember the rational planning of supports, so as to ensure the highest possible rigidity of the workpiece in the clamping device.
- It should be emphasised that the tests were carried out on flat samples, as it was the most favourable solution to achieve the objectives of the study. For complex-shaped thin-walled structures, the stiffness is different and the deformation effects of cutting cannot be directly transferred from the flat elements’ examination.

## Figures and Tables

**Table 1:** Chemical composition and properties of EN AW-2024 T351 aluminium alloy [41,42].

Chemical Composition [%]												
Si	Fe	Mg	Cu		Mn	Zn		Cr	Zr+Ti	Ti	Other	Al
≤0.5	≤0.5	1.2–1.8	3.8–4.9		0.3–0.9	≤0.25		≤0.1	≤0.2	≤0.15	≤0.15	Rest
**Properties**												
Density ρ [g/cm^3^]		Young’s module E [GPa]		Tensile strength R_m_ [MPa]			Offset yield strength R_p0.2_ [MPa]			Brinell hardness [HB]		
2.78		73		469			324			120		

**Table 2:** Technical parameters of the cutters used [43,44].

Symbol	Kennametal 25A03R044B25SED14	Sandvik R216.33-16040-AC32U
Number of teeth, z	3	3
Working part diameter d, mm	25	16
Overall length L, mm	101	92
Maximum depth of cut a_pmax_, mm	14.6	32
Clamping part diameter d, mm	25	16

**Table 3:** Technological parameters corresponding to individual milling strategies.

Technological Parameters	Strategies							
	HPC	HPC + CM		HPC + HSC		HSC	HSC + CM	
		HPC	CM	HPC	HSC		HSC	CM
Depth of cut a_p_, mm	4.5	4.3	0.4	4.3	0.4	0.956; 0.4 *	0.956	0.4
Milling width a_e_, mm	18.75	18.75	12	18.75	12	12	12	12
Cutting speed v_c_, m/min	1000	1000	200	1000	1200	1200	1200	200
Feed per tooth f_z_, mm/tooth	0.1	0.1	0.02	0.1	0.02	0.02	0.02	0.02
Rotational speed n, rpm	12,732	12,732	3979	12,732	23,873	23,873	23,873	3979
Number of passes i	2	2	1	2	1	9; 1*	9	1

* Last pass.

**Table 4:** Technical specification of Tenmex TF-5-2x foil strain gauges [45].

Specification	Unit	Value
**Resistance R**	Ω	120 ± 0.2%
**Constant of the strain gauge k**	-	2.15 ± 0.5%

**Table 5:** Relative deformation ε obtained for samples in which the textured surface layer was not removed, the analysed cutting strategies and the tested relations between the milling direction and the rolling direction.

Relative Deformation ε [μm/m]		
Strategy	Transversal Strain Gauge	Longitudinal Strain Gauge
Relation of milling direction to rolling direction: perpendicular		
HPC	−203.62	−402.59
HPC + CM	−149.24	−256.17
HPC + HSC	−113.18	−193.28
HSC	−101.21	−175.40
HSC + CM	−115.33	−168.66
Relation of milling direction to rolling direction: parallel		
HPC	−169.90	−301.28
HPC + CM	−98.53	−198.15
HPC + HSC	−105.54	−159.45
HSC	−26.47	−58.61
HSC + CM	−25.98	−51.59

**Table 6:** Statistical verification of the results of relative deformations ε (α = 0.05)—without textured surface layer removal.

	Strategy	Test	F	F_cr_	Result	t	t_cr_	Result
Relation of milling direction to rolling direction: perpendicular								
1	HPC	-	-	-	-	-	-	-
2	HPC + CM	1–2	2.4482	6.3883	$\sigma_{1}^{2}=\sigma_{2}^{2}$	−49.7480	2.3060	u_1_ ≠ u_2_
3	HPC + HSC	2–3	1.1101		$\sigma_{1}^{2}=\sigma_{2}^{2}$	−27.3148		u_1_ ≠ u_2_
4	HSC	3–4	1.7156		$\sigma_{1}^{2}=\sigma_{2}^{2}$	−8.5099		u_1_ ≠ u_2_
5	HSC + CM	4–5	1.0572		$\sigma_{1}^{2}=\sigma_{2}^{2}$	−3.7896		u_1_ ≠ u_2_
Relation of milling direction to rolling direction: parallel								
1	HPC	-	-	-	-	-	-	-
2	HPC + CM	1–2	1.2253	6.3883	$\sigma_{1}^{2}=\sigma_{2}^{2}$	−43.4807	2.3060	u_1_ ≠ u_2_
3	HPC + HSC	2–3	1.5196		$\sigma_{1}^{2}=\sigma_{2}^{2}$	−15.3339		u_1_ ≠ u_2_
4	HSC	3–4	3.3243		$\sigma_{1}^{2}=\sigma_{2}^{2}$	−45.1095		u_1_ ≠ u_2_
5	HSC + CM	4–5	1.0189		$\sigma_{1}^{2}=\sigma_{2}^{2}$	−4.6391		u_1_ ≠ u_2_

**Table 7:** Statistical verification of the results of relative deformations ε (α = 0.05)—with textured surface layer removal (CM).

	Strategy	Test	F	F_cr_	Result	t	t_cr_	Result
Relation of milling direction to rolling direction: perpendicular								
1	HPC	-	-	-	-	-	-	-
2	HPC + CM	1–2	3.6531	6.3883	$\sigma_{1}^{2}=\sigma_{2}^{2}$	−108.1382	2.3060	u_1_ ≠ u_2_
3	HPC + HSC	2–3	3.5439		$\sigma_{1}^{2}=\sigma_{2}^{2}$	78.6936		u_1_ ≠ u_2_
4	HSC	3–4	1.2329		$\sigma_{1}^{2}=\sigma_{2}^{2}$	4.1451		u_1_ ≠ u_2_
5	HSC + CM	4–5	1.9544		$\sigma_{1}^{2}=\sigma_{2}^{2}$	−26.4588		u_1_ ≠ u_2_
Relation of milling direction to rolling direction: parallel								
1	HPC	-	-	-	-	-	-	-
2	HPC + CM	1–2	2.4877	6.3883	$\sigma_{1}^{2}=\sigma_{2}^{2}$	−89.6754	2.3060	u_1_ ≠ u_2_
3	HPC + HSC	2–3	1.8990		$\sigma_{1}^{2}=\sigma_{2}^{2}$	69.3658		u_1_ ≠ u_2_
4	HSC	3–4	1.5648		$\sigma_{1}^{2}=\sigma_{2}^{2}$	−26.4235		u_1_ ≠ u_2_
5	HSC + CM	4–5	1.1690		$\sigma_{1}^{2}=\sigma_{2}^{2}$	−19.4783		u_1_ ≠ u_2_

**Table 8:** Statistical verification of the results of relative deformations ε (α = 0.05)—with textured surface layer removal (HSC).

	Strategy	Test	F	F_cr_	Result	t	t_cr_	Result
Relation of milling direction to rolling direction: perpendicular								
1	HPC	-	-	-	-	-	-	-
2	HPC + CM	1–2	1.5170	6.3883	$\sigma_{1}^{2}=\sigma_{2}^{2}$	−15.5511	2.3060	u_1_ ≠ u_2_
3	HPC + HSC	2–3	1.0658		$\sigma_{1}^{2}=\sigma_{2}^{2}$	5.3319		u_1_ ≠ u_2_
4	HSC	3–4	2.0656		$\sigma_{1}^{2}=\sigma_{2}^{2}$	37.6104		u_1_ ≠ u_2_
5	HSC + CM	4–5	2.6805		$\sigma_{1}^{2}=\sigma_{2}^{2}$	−37.3703		u_1_ ≠ u_2_
6	-	2–5	1.2176		$\sigma_{1}^{2}=\sigma_{2}^{2}$	2.3408		u_1_ ≠ u_2_
Relation of milling direction to rolling direction: parallel								
1	HPC	-	-	-	-	-	-	-
2	HPC + CM	1–2	1.2765	6.3883	$\sigma_{1}^{2}=\sigma_{2}^{2}$	25.4505	2.3060	u_1_ ≠ u_2_
3	HPC + HSC	2–3	1.1219		$\sigma_{1}^{2}=\sigma_{2}^{2}$	1.0162		u_1_ = u_2_
4	HSC	3–4	1.4325		$\sigma_{1}^{2}=\sigma_{2}^{2}$	−61.7903		u_1_ ≠ u_2_
5	HSC + CM	4–5	1.0406		$\sigma_{1}^{2}=\sigma_{2}^{2}$	24.9991		u_1_ ≠ u_2_

**Table 9:** Statistical verification of the results of relative deformations ε (α = 0.05)—comparison of pre-machining variants.

	Pre-Machining	Test	F	F_cr_	Result	t	t_cr_	Result
Relation of milling direction to rolling direction: perpendicular, strategy: HPC								
1	CM	-	-	-	-	-	-	-
2	HSC	1–2	1.5940	6.3883	$\sigma_{1}^{2}=\sigma_{2}^{2}$	−3.3418	2.3060	u_1_ ≠ u_2_
Relation of milling direction to rolling direction: perpendicular, strategy: HPC + HSC								
1	LACK	-	-	-	-	-	-	-
2	HSC	1–2	1.1684	6.3883	$\sigma_{1}^{2}=\sigma_{2}^{2}$	−1.2967	2.3060	u_1_ = u_2_
Relation of milling direction to rolling direction: parallel, strategy: HPC + CM								
1	LACK	-	-	-	-	-	-	-
2	HSC	1–2	1.2435	6.3883	$\sigma_{1}^{2}={\;\sigma }_{2}^{2}$	0.8170	2.3060	u_1_ = u_2_
Relation of milling direction to rolling direction: parallel, strategy: HPC + HSC								
1	CM	-	-	-	-	-	-	-
2	HSC	1–2	1.0253	6.3883	$\sigma_{1}^{2}=\sigma_{2}^{2}$	−3.9686	2.3060	u_1_ ≠ u_2_
Relation of milling direction to rolling direction: parallel, strategy: HSC								
1	LACK	-	-	-	-	-	-	-
2	HSC	1–2	1.3957	6.3883	$\sigma_{1}^{2}=\sigma_{2}^{2}$	6.2624	2.3060	u_1_ ≠ u_2_
